# A contrastive learning approach for ICU false arrhythmia alarm reduction

**DOI:** 10.1038/s41598-022-07761-9

**Published:** 2022-03-18

**Authors:** Yuerong Zhou, Guoshuai Zhao, Jun Li, Gan Sun, Xueming Qian, Benjamin Moody, Roger G. Mark, Li-wei H. Lehman

**Affiliations:** 1 Xi’an Jiaotong University, Xi’an, China; 2 Nanjing University of Science and Technology, Nanjing, China; 3 Chinese Academy of Sciences, Shenyang, China; 4 Institute for Medical Engineering & Science, Massachusetts Institute of Technology, Cambridge, MA 02139 USA

**Keywords:** Health care, Computer science

## Abstract

The high rate of false arrhythmia alarms in Intensive Care Units (ICUs) can lead to disruption of care, negatively impacting patients’ health through noise disturbances, and slow staff response time due to alarm fatigue. Prior false-alarm reduction approaches are often rule-based and require hand-crafted features from physiological waveforms as inputs to machine learning classifiers. Despite considerable prior efforts to address the problem, false alarms are a continuing problem in the ICUs. In this work, we present a deep learning framework to automatically learn feature representations of physiological waveforms using convolutional neural networks (CNNs) to discriminate between true vs. false arrhythmia alarms. We use Contrastive Learning to simultaneously minimize a binary cross entropy classification loss and a proposed similarity loss from pair-wise comparisons of waveform segments over time as a discriminative constraint. Furthermore, we augment our deep models with learned embeddings from a rule-based method to leverage prior domain knowledge for each alarm type. We evaluate our method using the dataset from the 2015 PhysioNet Computing in Cardiology Challenge. Ablation analysis demonstrates that Contrastive Learning significantly improves the performance of a combined deep learning and rule-based-embedding approach. Our results indicate that the final proposed deep learning framework achieves superior performance in comparison to the winning entries of the Challenge.

## Introduction

ICUs are designed to provide acute care for patients with severe and life-threatening injuries or illnesses using sophisticated bedside monitors such as pulse oximeter (PPG), electrocardiogram (ECG), arterial blood pressure (ABP) catheter, central venous pressure catheter and ventilators. Ideally, these monitors with a built-in alarm system can send an alert to the healthcare providers when a patient’s physiological signals are out of pre-defined ranges. On the one hand, arrhythmia alarms in the ICU based monitors are deliberately designed to be highly sensitive in order not to miss any life-threatening events. However, high sensitivity compromises the specificity of these alarms^1^. According to Drew et al.^2^ the false alarm ratio in ICUs can be as high as 88.8%.

Alarms are falsely triggered by many factors, including noise and artifacts from patient movement, power line interference, electrode contact noise, and data collecting device noise. Falsely triggered alarms become an unseen threat in ICUs for they not only lead to sleep deprivation^3^, inferior sleep structure^4^, stress for both patients and staff^5^ and depressed immune systems^6^, but also put patients at risk for the desensitization to warnings and slowing of response times. By contrast, only 2–9% of all ICU alarms are correctly triggered and these alarms do require an urgent and professional response^7^. Therefore, false alarms present an important problem in ICUs today^8^.

Methods proposed to reduce the rate of false arrhythmia alarms in the PhysioNet 2015 Challenge^9^ can be roughly divided into two categories: rule-based methods and machine learning methods. The best-performing methods from the Challenge 2015 are mostly rule-based and require hand-engineered feature crafting. Rule-based methods mainly use expert-defined rule-based logic analysis to analyze patients’ physiological signals^10^. Specifically, methods in this field mainly consist of signal quality evaluating and QRS-complex detection in order to analyze heart rate. Machine learning algorithms have been widely used in the medical field^11,12^. Peng et al. introduce machine learning techniques to transform the nuclear magnetic resonance (NMR) correlational map into user-friendly information for point-of-care disease diagnostic and monitoring^11^. Lau et al. utilize polymerization studies and two dimensional-nuclear magnetic resonance spectrometry (2D-NMR) to investigate the hypothesis that HOCl oxidation alters fibrinogen conformation and T2 relaxation time of water protons in the fibrin gels^12^. In the false alarm reduction problem, conventional machine learning based methods train a classification model using hand-crafted features as input to classify the alarms^13^. The performance of these prior methods depends highly on the quality of these hand-crafted features or on the design of rules which cannot automatically model the complex patterns in waveform data.

Although deep learning provides powerful representation learning techniques to automatically capture complex patterns in the data, conventional deep learning approaches for physiological waveform analysis in false alarm reduction have had limited success in out-performing the rule-based techniques in the PhysioNet 2015 Challenge^9^. False arrhythmia alarm reduction in ICUs is a challenging problem for deep learning approaches due to the high-dimensional data from long sequence length of the multi-channel physiological waveform signals, imbalanced classes of true vs. false alarms, and most importantly, a limited number of records with ground-truth labels due to the fact that expert-annotation of arrhythmia alarms is laborious and costly to obtain.

To address the above challenges, we design a novel deep contrastive learning framework to detect true arrhythmia alarms based on a CNN^14^ architecture. In the proposed model, we use CNN as the signal encoder to automatically extract the features of the input signals relevant for our classification task. We propose to use the idea of Contrastive Learning with Siamese network^15^ and discriminative constraints to learn an effective lower-dimensional representation of high-dimensional waveform signals to improve the signal encoder, prevent over-fitting and overcome the problem of insufficient training data. In addition, to leverage all available training records in the dataset, we train our deep learning models using records from all alarm types simultaneously, and use learned embeddings from records’ alarm types as input to our deep learning models to enable classification across multiple alarm types at the same time. Finally, we augment our model by using a rule-based approach to learn an embedding as input to our deep models. This enables our technique to achieve label-efficient learning in a small labeled data setting by leveraging rule-based techniques that utilize known physiological structure of the signals for the classification task. Results on the unseen test set show that our method outperforms all submitted methods on the real-time event in the PhysioNet Challenge 2015^9^.

Our main contributions are summarized as follows:

- We propose a deep learning model in false alarm reduction, using CNN as the signal encoder to reduce the length of input signals and detect temporal and spatial patterns in multi-channel waveform data.
- We develop a Contrastive Learning framework by using Siamese Network and calculating a discriminative constraint to prevent over-fitting and address the challenge of limited training data.
- We augment our deep learning model with embeddings generated by a rule-based method to leverage domain-knowledge specific to each alarm type for label-efficient representation learning.

## Related work

### Rule-based methods

Plesinger et al. test each channel in the record for regular heart activity using the QRS distribution and derived R-R information^10^. Daluwatte et al. develop an algorithm based on global heartbeat annotations generated by fusing individual heartbeat detection from multiple physiological signals and then apply an arrhythmia criterion to the global heartbeat detection to classify the alarm^16^. Ansari et al. use a multi-modal peak detection algorithm and combines the results from several peak detection algorithms to create a robust peak detection algorithm^17^. Tsimenidis et al. propose a method that includes high-pass filtering to remove baseline instability, scaling to normalize waveform amplitudes, detection of noisy and flat waveforms, differentiation to accentuate sharp waveform edges, beat detection, timing between beats preceding alarm onset, and detection of alarm conditions^18^. He et al. use a derived signal quality index (SQI) to reveal the degree of signal quality^19^. The SQI-weighted residual error of Kalman filters (KF) is used to complete the data fusion for evaluating the heart rate (HR). Finally, the algorithm of arrhythmia false alarm reduction is developed based upon the method of combining SQIs and HR estimations derived from ECG and ABP waveforms. Fallet et al. estimate heart rate from pulsatile waveforms using an adaptive frequency tracking algorithm or computed from ECGs using an adaptive mathematical morphology approach based on the quality of available signals^20^. Furthermore, they introduce a supplementary measure based on the spectral purity of the ECGs to determine if a ventricular tachycardia or flutter/fibrillation arrhythmia has taken place. Finally, alarm veracity is determined based on a set of decision rules on heart rate and spectral purity values. Couto et al. use simultaneous ECG and pulsatile waveforms^21^. QRS detectors are used to produce for each signal a set of QRS detections which are to be used for detecting false alarms. In case some of the signals may be noise-contaminated, the signal quality of each waveform is evaluated to determine whether the QRS detection obtained on that waveform is reliable. A set of rules is then used for each alarm type. Although rule-based methods are effective and commonly used in medical fields, extensive expert knowledge is needed to design the rules and evaluate.

### Traditional machine learning methods

Antink et al. present an approach that analyzes multi-modal cardiac signals in terms of their beat-to-beat intervals as well as their average rhythmicity^22^. Based on this analysis, several features in time and frequency domain are extracted and used for several machine learning approaches. Eerikainen et al. train Random Forest classifiers for every type of arrhythmia with arrhythmia-specific features computed from signal quality information and physiological features^23^. Kalidas et al. use a combination of logical analysis and SVM-based machine learning techniques^13^. Information from original signals is used for logical analysis and to form the features set. Caballero et al. develop a decision tree for each arrhythmia category, which is combined with domain knowledge to produce a set of if/else statements^24^. Using the ABP and PPG signals, separate decision trees are trained. Afghah et al. propose a model based on coalition game theory that considers the inter-features dependencies in determining the salient predictors with respect to false alarms^25^. Antink et al. present an approach that analyzes multi-modal cardiac signals in terms of their beat-to-beat intervals as well as their average rhythmicity^22^. Based on this analysis, several features in time and frequency domains are extracted and used for subsequent machine learning tasks. Zaeri-Amirani et al. propose a low-computational complexity game-theoretic feature selection method which is based on a genetic algorithm that identifies the most informative biomarkers across the signals collected from various monitoring devices^26^. Au-Yeung et al. applies a Random Forest and meanwhile performed feature selection in order to reduce the complexity of the models and improve the efficiency of the algorithm^27^.

### Deep learning methods

Lehman et al. present a supervised generative model to classify ventricular tachycardia alarms using non-linear embeddings of ECG dynamics^28^. The model is a variant of a Denoising Autoencoder, learned using a combination of discriminative and generative loss. Furthermore, feature transformations are explored by utilizing known physiological structure within ECG signals to enable learning under the constraints of limited labeled data. To this end, a multi-stage approach is proposed to utilize the FFT-transform of consecutive heart beats. Hooman et al. present a method for training neural networks based on neuroevolution by utilizing the Dispersive Flies Optimisation algorithm in a gradient-free population-based scheme^29^. Mousavi et al. propose a deep learning-based network composed of the CNN layers, attention mechanism, and LSTM units to reduce false alarm arrhythmia in ICUs^30^. Yu et al. design a multi-channel deep group convolutional neural network for false alarm reduction^31^. Their model takes multi-channel raw signals as input and different kernels are used for convolution operation according to the type of alarm. Zihlmann et al.^32^ propose two deep learning models for classification of arbitrary-length ECG recordings using the Physionet Challenge 2017^33^ dataset. The first model is a deep CNN architecture with averaging-based feature aggregation across time. The second model combines convolutional layers for feature extraction with long-short term memory (LSTM) layers for temporal aggregation of features. They use two data augmentation techniques, dropout bursts and random resampling, for ECG data during training procedure. Hong et al. propose an ensemble classifier to combine expert features and deep learning models together for ECG classification^34^ on Physionet Challenge 2017 dataset. Hyvarinen et al. propose a learning principle for unsupervised representation learning on time series^35^, which is based on analyzing nonstationarity in temporal data by discriminating between time segments. Kiyasseh et al. propose a family of self-supervised pretraining mechanisms based on contrastive learning for physiological signals^36^. Pei et al. propose a model for time series analysis that learns a similarity measure over pairs of time series in a supervised manner^37^. In the Siamese Network, two time series are inputted to the same recurrent network for feature extraction. Wu et al. propose an end-to-end deep learning model to learn local representations of time series^38^. A local embedding loss is applied to optimize a Siamese Network and a feature space that preserves the temporal location-wise distances between time series can be learned in their framework.

## Methodology

In deep learning, CNN has been successfully applied in many different domains such as image classification^39^ as well as different natural language processing tasks^40,41^. Motivated by the success of CNN and its variants in these various domains, researchers have started using CNN for time series classification^42^. Commonly used CNN models such as FCN^43^ and ResNet^44^ are good at extracting local spatial features and ResNet can support a very deep architecture. However, in order to take advantage of the power of FCN and ResNet, these models use as many CNN layers as possible. It is hard for us to train since we have a limited number of training records. Therefore, we train a deep learning model to classify the arrhythmia alarms using a 1-dimensional CNN which can extract local features of 1-dimensional data such as time series data. It is small and easy to train. In the proposed model, four CNN blocks are used to extract the features of the raw input signals. Each block has a different kernel size. In order to improve the performance of the feature extractor and to avoid the overfitting problem, which is crucial in this problem, we propose a pair-wise loss function which utilizes contrastive learning. While other approaches use contrastive learning between different records, we utilize contrastive learning method and compute pair-wise loss between two different segments inside the same alarm record. Specifically, the proposed Siamese Network architecture learns latent representations of the signals through contrastive learning from two segments of the same patient waveform record, namely, the ‘alarm-trigger signal’ (or ‘alarm signal’ for short), which is the waveform segment that triggered the alarm, and a pre-alarm ‘baseline signal’ which is a randomly sampled waveform segment of the same length representing the baseline of the same patient prior to the alarm-triggering event. In addition, in order to leverage the domain-knowledge encoded in the rule-based methods, we augment our model with output from the rule-based method proposed by Plesinger et al.^10^. For each record, we feed the output of the rule-based method into our networks to generate an embedding. After converting raw signals into a representation vector, it is then concatenated with the alarm-type embedding and the rule-based embedding as input to a classification layer to generate the probability of a false alarm for each record. At last, the study was performed in accordance with the relevant guidelines and regulations and in accordance with the Declaration of Helsinki.

### Model architecture

**Figure 1:**
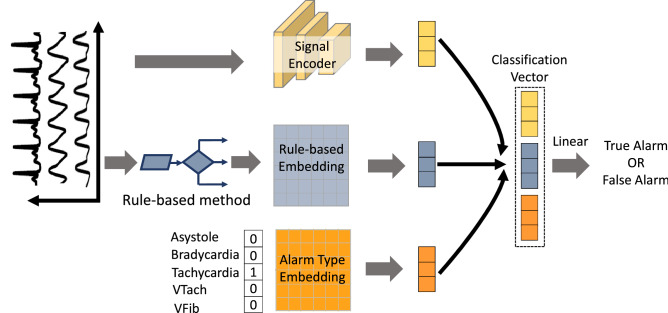
Illustration of the proposed model architecture and deep learning framework. Figure generated in PowerPoint version 1808, https://www.microsoft.com.

Figure 1 illustrates the proposed model architecture and deep learning framework. The architecture includes a signal encoder, two fully connected layers and a classification layer. The signal encoder is based on a CNN architecture, and hence, does not rely on heavy hand-crafting of feature engineering. We denote the number of time steps as $T_{l}$, the number of variables as $M_l$, the kernel size of CNN layer as $D_l$, the input as $X^{(l)} \in {\mathbb {R}}^{M_{l} \times T_{l}}$, the full sizes of filters as $W^{(l)} \in {\mathbb {R}}^{M_{l-1} \times D_{l} \times M_{l}}$, and the bias as $B^{(l)} \in {\mathbb {R}}^{M_{l} \times T_{l}}$ in the $l{\text{th}}$ CNN layer. Let $X_{m,t}^{(l)}$ be the value of the $m{\text{th}}\, (0 < m \le M)$ variables with the $t{\text{th}} \,(0 < t \le T)$ time step input series. By the activation function $f(\cdot )$ Rectified Linear Unit(ReLU), we can get the value of each position for $\forall {m} \in \lbrace 1,2,3,\ldots ,M_{l}\rbrace , \forall {t} \in \lbrace 1,2,3,\ldots ,T_{l}\rbrace , \forall {l} \in \lbrace 1,2,3,\ldots ,L\rbrace$.

1 $$\begin{aligned} X_{m,t}^{(l)}=f\left( B^{(l)}_{m,t} + \sum _{j=0}^{D_l-1}\sum _{i=0}^{M_l-1} W_{i,j,m}^{(l)} X_{i,t+j}^{(l-1)} \right) . \end{aligned}$$

Two fully connected layers are the alarm embedding layer and rule-based embedding layer respectively. With different alarm types, signals may have different features and characteristics unique to each alarm type in distinguishing between a true vs. a false alarm. Since we already have the information about the triggered alarm type of each record in the training datasets, alarm type of each sample can provide useful information for the model when classifying. The alarm embedding layer converts the alarm type of a sample to an embedding with a fixed size. Given a one-hot vector $A \in {\mathbb {R}}^{1\times N}$ as an alarm type of a given record, the alarm embedding layer transforms it into:

2 $$\begin{aligned} E_{a}=AW_{a}, \end{aligned}$$

where $W_{a} \in {\mathbb {R}}^{N \times S_{a}}$ is a trainable parameter of the alarm embedding layer, *N* indicates the number of arrhythmia alarm types and $S_{a}$ indicates the size of alarm embedding.

The rule-based embedding layer converts the output by a rule-based method into an embedding. We denote the result output by the rule-based method as *R*. The rule-based embedding layer transforms it into:

3 $$\begin{aligned} E_{r}=RW_{r}, \end{aligned}$$

where $E_{r}$ is the embedding of the rule-based output, $W_{r} \in {\mathbb {R}}^{S_{r}}$ is a trainable parameter of the rule-based embedding layer and $S_{r}$ indicates the size of the rule-based embedding.

We combine the strong learning ability of deep learning models and clinical rules and experiences from rule-based method by concatenating the latent encoding of raw signals $E_e$, the alarm type embedding $E_{a}$ and the rule-based embedding $E_r$ as follows:

4 $$\begin{aligned} E=E_{e}\oplus E_{a}\oplus E_{r}. \end{aligned}$$

Finally the concatenated vector $E\in {\mathbb {R}}^{1\times (S_e+S_a+S_r)}$ is fed into the classification layer, which is comprised of a fully-connected layer followed by a sigmoidal output layer to get the output probability *O* of the triggered alarm being true:

5 $$\begin{aligned} O=\sigma (EW_c), \end{aligned}$$

where $W_c \in {\mathbb {R}}^{(S_e+S_a+S_r)\times 1}$ is the trainable parameter of the classification layer and $\sigma$ is the sigmoid function.

**Figure 2:**
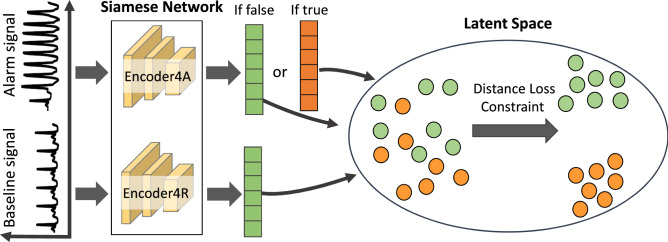
We use the idea of Siamese Network to calculate our discriminative constraint. Here, Encoder4A and Encoder4R take an ‘alarm signal’ and a ‘baseline signal’ sampled from the same waveform record as inputs and output their feature vectors respectively. The distance loss is then used to find the similarity of the inputs by comparing their feature vectors. The ’alarm signal’ refers to the multi-channel waveform segment that triggered the alarm (e.g., 10-s segment prior to the alarm onset), and the ’baseline signal’ segment is randomly sampled from a prior time interval of the same record. Figure generated in PowerPoint version 1808, https://www.microsoft.com.

### Loss function

In the false alarm reduction problem, the label of each record is just TRUE and FALSE, while the length of each waveform channel of each record is 75,000 samples (5-min segment sampled at 250 Hz), which means the supervised information is too small to train a deep learning model. Therefore, instead of only using the label of each record, we design a pair-wise similarity-based loss function calculated by using different segments in the waveforms of the same record as additional information to train our model. The choice of similarity-based loss is motivated by the fact that, clinically, the detection of a true VT event often involves comparing the signals immediately prior to the alarm onset with signals from the same patient at an earlier time point to determine whether there has been a change in the patient’s ECG from his or her baseline. Additionally, sampling from the same patient’s baseline signals functions as a data augmentation scheme to increase our effective sample size to improve performance in a small labeled data setting. This similarity-based loss works as a discriminative constraint and is combined with the binary entropy loss during training. Our model takes the 10 s sequence of the multi-channel waveforms immediately prior to the alarm onset as the ’alarm signals’. We use alarm signals instead of the whole signals for classification since the exact time of the event that triggered the alarm is within 10 s of the alarm^45^. Using the alarm signals can effectively reduce the computational complexity and improve the accuracy of classification due to the difficulty of very long time series classification. Meanwhile, we also randomly sample a sequence with the same length as random baseline signals prior to time $t-10$ s from the same patient as the pre-alarm ‘baseline signal’, where *t* is the alarm onset time. The signal encoder is a Siamese Network, which means it can be seen as two identical encoders, Encoder4A and Encoder4R. They have the same configuration with the same parameters and weights. Alarm signals and baseline signals are fed into Encoder4A and Encoder4R respectively to get their corresponding feature vector $E_e^{R}$ and $E_e^{A}$. These two feature vectors are then used for calculating the discriminative constraints. Figure 2 illustrates the Siamese Network architecture in calculating the discriminative constraint. The discriminative constraint of a record depends on the ground-truth of its alarm. If the triggered alarm is false, then the feature vector of its baseline signals $E_e^{R}$ should be close to the feature of the alarm signals $E_e^{A}$ since the monitoring system misjudged the vital signs of the last 10 seconds by mistakenly triggering an alarm and these two feature vectors should be considered as a similar representation. Their constraint can be defined as follows:

6 $$\begin{aligned} C_{false}^{(i)}=-\log (\sigma ({f(E^{A})}^{T}f(E^{R}))), \end{aligned}$$

where $C_{false}^{(i)}$ is the discriminative constraint of a record with a false alarm in a mini-batch. $\sigma$ is the sigmoid function. $f(\cdot )$ is the signal encoder. If the alarm is true, $E_e^{R}$ should be distant from $E_e^{A}$ for the alarm signals to represent real abnormal vital signs while baseline signals do not. Then the constraint should be defined as:

7 $$\begin{aligned} C_{true}^{(j)}=-\log (\sigma ({-(f(E^{A})}^{T}f(E^{R})))), \end{aligned}$$

where $C_{true}^{(j)}$ is the discriminative constraint of a record with a true alarm in a mini-batch. The discriminative constraint in a mini-batch can be calculated as:

8 $$\begin{aligned} C=\frac{1}{N_{1}}\sum C_{false}^{(i)}+\frac{1}{N_{2}}\sum C_{true}^{(j)}. \end{aligned}$$

In the above equation, *C* is the calculated discriminative constraint in a mini-batch. $N_{1}$ is the number of false alarm records in a mini-batch and $N_{2}$ is the number of true alarm samples.

In the training procedure, we try to minimize the discriminative constraint for each mini-batch. Meanwhile, we also use binary cross entropy loss(BCE) to ensure that the classification part of the model can correctly classify the classification vector of input signals. The binary cross entropy loss is calculated as:

9 $$\begin{aligned} L_{B C E}=\frac{1}{N} \sum _{i}^{N}-y_{i}\log {\widehat{y}}_{i}-\left( 1-y_{i}\right) \log \left( 1-{\widehat{y}}_{i}\right) . \end{aligned}$$

In the above equation, $L_{B C E}$ is the calculated binary cross entropy loss. *N* is the number of alarm records in a mini-batch. $y_{i}$ is the label of the triggered alarm of a sample. ${\widehat{y}}_{i}$ is the probability of the alarm of a sample being true.

The loss function we minimize during training is the combination of discriminative constraint and the binary cross entropy loss. We formulate the loss function as the weighted sum of a binary cross entropy loss and the discriminative constraint as:

10 $$\begin{aligned} L=L_{B C E}+w*C, \end{aligned}$$

where *w* is the weight.

## Experiments

### Datasets

The study uses publicly-available, de-identified dataset from PhysioNet^9^. The PhysioNet/Computing in Cardiology Challenge 2015 provides a dataset with 750 records for algorithm development and 500 unrevealed records. These records consist of patients’ physiological signals that have been collected from four hospitals in the United States and Europe, sourced from the devices made by three major manufacturing companies of intensive care monitor devices^9^. Each record contained an alarm for one arrhythmia event and the triggered alarm was reviewed and labeled by a team of expert annotators to either true or false. Asystole (ASY), Extreme Bradycardia (EBR), Extreme Tachycardia (ETC), Ventricular Fibrillation or Flutter (VFB), or Ventricular Tachycardia (VTA) are the five alarm types in the datasets. Each record contained two leads of ECG, and at least one pulsatile waveform of either PPG or ABP. In some records, both pulsatile waveforms or a respiratory signal were present. All signals have been re-sampled to a resolution of 12 bits and had a sampling frequency of 250 Hz, therefore each record is 5 min or 5 min and 30 s long. The alarm onsets are 5 min from the beginning of each record. The exact time of the event that triggered the alarm varies somewhat from one record to another, but in order to meet the AAMI standards, the commencement of the event must be within 10 seconds of the alarm^45^. For Event 1, which is a real-time alarm suppression problem, each record is exactly 5 min long. For Event 2, each record contains an additional 30 s of signals following the time of the alarm. We focus on Event 1 in this paper. Some statistics are shown in Table 1, and more detailed statistics about this dataset could be found in Supplementary Appendix.

**Table 1:** Statistics of the PhysioNet Challenge 2015 training set.

Arrhythmia	Definition	Count/ratio	True alarms	False alarms
ASY	0 beats in 4s	122/17%	22	100
EBR	> 5 beats, HR < 40 bpm	89/11%	43	46
ETC	> 17 beats, HR > 140 bpm	140/17%	131	9
VTA	> 5 ventricular beats, HR > 100 bpm	341/47%	89	252
VFB	Fibrillation waves in 4 s	58/7%	6	52

### Pre-processing

In this paper, we focus on the real-time event and only use the first 300 s of signals for each record, which means only information prior to the alarm onset is used. Therefore, for each record we use, the event that triggered the alarm is during the last 10 s immediately prior to the alarm onset. Before we feed the signals into the model, raw signals are subjected to imputation and standardization. In the imputation part, some patients do not have the record of certain signals. Therefore, these missing signals are imputed with 0. In the standardization part, each signal is normalized to a range of 0 to 1.

### Setup

In the experiments, we use fivefold cross validation. For each cross validation, one fold that is used for evaluating the model has 150 records and the remaining fourfold that are used to train the model has 600 records. In the end, all evaluation results are averaged.

We use 4 parallel CNN blocks in the signal encoder. These 4 CNN blocks have different filter sizes, which are 50, 100, 200, and 400 respectively. Each CNN block has two convolutional layers with the same filter size. The first convolutional layer is composed of 64 filters with a stride of 5, a Batch Normalization layer^46^ and a Rectified Linear Unit(ReLU) layer^47^. The second convolutional layer has the same hyperparameters as the first layer except for the number of input channels. Convolutional layers are followed by a Global Max Pooling layer to aggregate high-level discriminative features and flatten features across channels. The output sizes of the rule-based embedding layer and the alarm type embedding layer are both set as 64.

Our model was trained with a maximum of 1000 epochs and a mini-batch size of 256. We use Adam optimizer^48^ to minimize the BCE loss and the discriminative constraint with learning rate of 0.001. To prevent the over-fitting problem, we use L2 regularization with a value of 0.0005. The dropout rate before each CNN block is set as 0.8. To overcome the problem of imbalanced classes, a weight of 1.5 was added to the positive samples in the BCE loss function as the number of negative alarm records is roughly 1.5 times than the number of positive alarm records. The weight of the discriminative constraint is set to 1.5 during training. The rule-based method with which we choose to combine our model is proposed by Ref.^10^. Our model was implemented using Python programming language and PyTorch deep learning library^49^.

### Compared methods

We evaluate our proposed model on the hidden test set and present comparisons to the existing deep learning methods commonly used for time-series classification and the top three methods of the real-time event listed on the challenge website. The compared methods are summarized as follows. (1) *MLP* We apply the multi-layer perceptron as a feature extractor of the input waveform and then use a dense layer to classify. (2) *FCN* We use a fully-connected convolutional network as the feature extractor of the input waveform. (3) *ResNet* We use ResNet as the feature extractor of the input waveform (4) *RB1* A rule-based method based on descriptive statistics and Fourier and Hilbert transforms^10^. (5) *ML1* A machine learning model that uses a combination of logical and SVM-based techniques^13^. (6) *RB2* A rule-based method that detects QRS and analyses the signal quality and then applies a different rule to each alarm type^21^. (7) *EDGCN* A deep group convolutional neural network proposed by Yu et al.^31^.

**Table 2:** Performance comparison on the test set of PhysioNet Challenge 2015.

Arrhythmia	MLP			FCN			ResNet			RB2			ML1			RB1			EDGCN			Ours		
	TPR (%)	TNR (%)	Score	TPR (%)	TNR (%)	Score	TPR (%)	TNR (%)	Score	TPR (%)	TNR (%)	Score	TPR (%)	TNR (%)	Score	TPR (%)	TNR (%)	Score	TPR (%)	TNR (%)	Score	TPR (%)	TNR (%)	Score
ASY	17	82	53.95	61	78	64.48	83	64	61.68	75	94	82.46	75	90	78.95	100	97	97.06	78	82	73.68	100	97	97.42
EBR	92	9	37.61	67	64	42.28	87	41	49.57	96	63	72.06	92	84	77.78	100	74	84.38	100	71	82.47	100	72	83.51
ETC	100	0	87.80	100	0	95.50	100	0	95.50	100	80	98.63	100	80	98.63	97	100	87.65	100	60	98.20	97	100	87.80
VTA	21	87	44.40	15	85	41.18	56	69	48.55	71	95	73.26	89	90	75.10	82	84	72.73	90	80	75.91	91	83	78.75
VFB	0	96	50.00	56	98	71.62	78	90	77.27	83	94	84.09	100	71	75.00	83	91	81.82	100	92	93.10	78	94	80.30
Real-time	66	77	51.54	66	80	52.79	83	66	59.11	89	**91**	79.02	94	82	79.44	93	87	81.62	96	80	80.68	**96**	86	**84.47**

Best performing values in each performance metric are given in bold.

### Results

The evaluation metrics for false alarm reduction are classification accuracy (ACC), true positive rate (TPR) and true negative rate (TNR). The PhysioNet Challenge 2015^9^ also provides an official scoring mechanism for evaluating. It is defined as $score = (TP+TN)/(TP+TN+FP+5 \times FN)$, where *TP* is true positives, *FP* is false positives, *FN* is false negatives, and *TN* is true negatives. The Challenge Score focuses more on the TPR value, since mistakenly classifying a true alarm as false results in significantly more severe consequences.

During training, we evaluate our model on the fivefold validation set. The average of the challenge score from the fivefold result is 87.00 with a standard deviation of 4.84. The detailed results are included in Supplementary Appendix.

Table 2 shows the detailed performance comparisons with the compared methods on the hidden test set. The performance of each alarm type is denoted as N/A since there are no such statistics in the paper. The higher the ACC, TPR, TNR and score, the better the performance. It is observed that our proposed contrastive learning model out-performs other baseline methods in the hidden test set. Note that the baseline methods in Table 2, including FCN and ResNet, are trained *without* contrastive learning. In Supplementary Appendix, we also present the performance comparison of FCN, ResNet and our CNN as different “backbone” encoders in our proposed contrastive learning framework.

Comparison with other models, especially with rule-based models, shows that it is difficult for common deep learning models to achieve high performance. There are many reasons for the poor performance of these models. First of all, many possible reasons can lead to false alarms including noise, patient manipulation or movement, mis-configuring, staff manipulation and leads falling off or mis-identification of signals. Second, the very long sequence length and imbalanced classes in the given datasets are big challenges for deep learning models. In addition, the number of labeled samples is crucial for classification using deep learning models while there are only 750 samples in the given training set which extremely limits the performance of these models. In our proposed model, we use 4 different kernel sizes from 50 to 400 in the CNN layers, which help alleviate the problem that the sequence length is too long for larger kernel sizes and can more effectively detect abnormal signals.

#### Ablation study

Our method has three components: signal encoder, discriminative constraint and rule-based embedding. We implement an ablation study by analyzing the quantitative results on the hidden test set as shown in Table 3. It can be observed that the more components we use the higher the performance is. Using only the signal encoder, the real-time event score can only achieve a challenge score of 60.35, which is only slightly better than the performance of ResNet, a common deep learning model with the best score in Table 2.

**Table 3:** Quantitative results of ablation study on test set.

Components			ASY	EBR	ETA	VTA	VFB	Real-time
Basic	Rule	Constraint						
$\checkmark$			74.85	40.43	95.50	51.69	52.13	60.35
$\checkmark$		$\checkmark$	62.30	46.62	**96.40**	56.63	55.56	60.69
$\checkmark$	$\checkmark$		96.13	80.20	95.50	69.93	**80.30**	78.90
$\checkmark$	$\checkmark$	$\checkmark$	**97.42**	**83.51**	87.80	**78.75**	**80.30**	**84.47**

Best performing challenge scores in each column are given in bold.

We observe that using discriminative constraints during training leads to improved performance in the overall real-time event score. Furthermore, it improves the performance of all alarm types except asystole. Augmenting the CNN-based signal encoder with a rule-based embedding can improve the performance significantly. The rule-based model contains information about the descriptive statistics and fuzzy logic derived based on domain-knowledge of the waveform for each alarm type, and thus enhances the signal encoder’s ability to more accurately distinguish between true vs. false alarms.

The discriminative constraints utilize the idea of contrastive learning to address the problem of over-fitting and imbalanced classes. Our results indicate that adding discriminative constraints to the combined a model of signal encoder and rule-based embeddings leads to the best performance in the Challenge score, with significant performance improvement from 78.90 to 84.47. Our results demonstrate the effectiveness of the proposed loss function.

## Conclusions and future work

False arrhythmia alarm reduction in ICUs is a challenging task for deep learning due to the very long sequence length of physiological signals, imbalanced classes and a limited number of labeled records. In this paper, we present a contrastive learning framework based on Siamese Network for false alarm reduction. During training, we use discriminative constraints to improve the feature extraction of signals. Furthermore, we augment our proposed model with a rule-based technique by using embeddings from the outputs of the rule-based method to regularize our deep learning model for label-efficient representation learning. Results show that the proposed method detected 86% of false alarms in the test set. The detection rate of true alarms was 96%. Using the official given scoring equation of the challenge, we achieved a score of 84.47 in the real-time event, outperforming other methods in the same task in the 2015 PhysioNet Challenge for the false arrhythmia alarm reduction.

Since the supervised information is too small to train a common deep learning model, making better use of the input data may be a promising direction. In future work, we will consider using self-supervised learning techniques to expand the scale of training data since we can set multiple pseudo-labels according to the downstream task to pretrain the model. Another potential direction is to pretrain an unsupervised learning model, such as BERT for the unlabeled time series data and then finetune the model in the downstream task.

## Supplementary Information

Supplementary Information.

Supplementary Figure 1.
