# Supplementary material for: A contrastive learning approach for ICU false arrhythmia alarm reduction
Source: Sci Rep. 2022 Mar 18;12:4689. doi: 10.1038/s41598-022-07761-9 (PMC8933571; doi:10.1038/s41598-022-07761-9)
Supplement: Supplementary file 1 — Supplementary Information. [file 41598_2022_7761_MOESM1_ESM.pdf]

Appendix

Signal encoder We evaluate several commonly used deep learning models, FCN, ResNet, Transformer and our CNN, as "backbone" encoders for our proposed contrastive learning framework. Results on the validation set are listed in Table 1. All models are trained using binary cross entropy loss combined with discriminative constraint and use rule-based embedding. Results show that our CNN has the best performance in the validation set among these three models. FCN excludes any pooling operation in its CNN blocks. Therefore, input signals maintain the same length during forward propagation. By comparison, our CNN has different kernel sizes and reduces the length of input waveform during forward propagation, which helps reduce the spatial size of the representation, the amount of parameters and computation in the network. Although ResNet can increase the depth of the network and reduce the effect of vanishing gradient problem, the large number of parameters causes the model to be very slow in training and in making predictions. The results of ResNet and Transformer as the backbone model show that latest or large-scale deep learning models may be not suitable for the false alarm reduction problem due to the limited number of training records and a large number of parameters of these models. In the main text of this paper, we present the hidden test set performance of our proposed contrastive learning framework using our CNN, the best performing model from validation data.

Table 1. Performance of our proposed framework using different backbone encoders on validation set.

Arrhythmia	FCN			ResNet			Transformer			Our CNN		
	TPR	TNR	Score	TPR	TNR	Score	TPR	TNR	Score	TPR	TNR	Score
ASY	95%	97%	93.65	95%	94%	91.27	96%	93%	90.30	95%	95%	92.06
EBR	98%	84%	87.10	96%	81%	81.44	100%	75%	97.71	100%	81%	91.01
ETC	100%	22%	95.00	100%	78%	98.57	100%	75%	97.71	100%	44%	96.43
VTA	82%	87%	72.35	83%	85%	72.07	78%	84%	70.56	91%	88%	80.97
VFB	83%	90%	83.87	100%	94%	94.83	100%	92%	92.88	83%	90%	83.87
Score	93%	**88**%	81.98	94%	**88**%	82.27	95%	85%	81.55	**97**%	**88**%	**87.00**

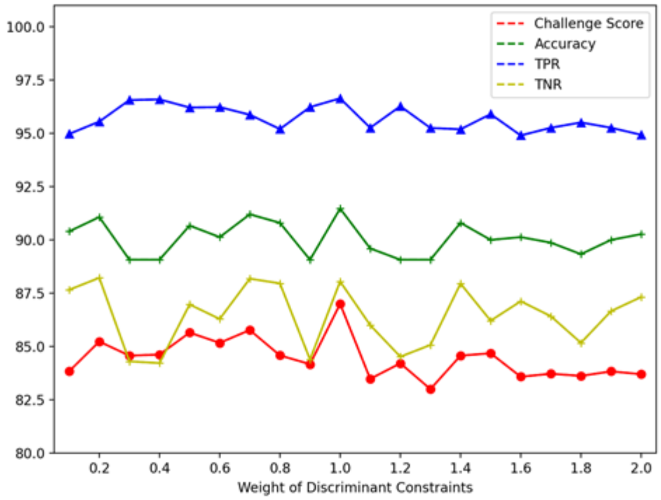

Figure 1. Quantitative results of different weights of Discriminant Constraints on the validation set.

Table 2. Quantitative results of different hidden feature sizes on the validation set.

Feature size	Challenge Score	Accuracy	TPR	TNR
16	83.75	89.47%	95.51%	85.59%
32	84.64	90.00%	95.88%	86.15%
64	**87.00**	**91.47**%	**96.64**%	88.05%
128	85.90	91.33%	95.91%	**88.34**%

Table 3. Performance on the 5-fold validation set

Kth fold	Challenge Score	Accuracy	TPR	TNR
1st	88.31	90.67%	98.41%	85.06%
2st	84.57	91.33%	94.74%	89.25%
3st	86.08	90.67%	95.92%	88.12%
4st	95.33	95.33%	100.00%	92.47%
5st	80.72	89.33%	94.12%	85.37%
Average	87.00	91.46%	96.64%	88.05%
Standard deviation	4.84	2.04	2.23	2.72

Table 4. Number of records with each signal type in training set.

Name	I	II	III	V	aVL	aVR	aVF	MCL	PPG	RESP	ABP
Count	13	728	39	684	2	3	3	28	627	278	343
Ratio	1.7%	97%	5.2%	91.2%	0.27%	0.4%	0.4%	3.7%	83.6%	37%	45.7%

Hyperparameters We test the choice of the weight of discriminant constraints and the hidden feature size of the encoder. For the choice of the weight of Discriminant Constraints, we have tested the Challenge Score, Accuracy, TPR and TNR of models trained using different weights of the Discriminant Constraints. Weights start at 0.1 and end at 2.0, with a step of 0.1. From Figure 1, we can see that the model achieves the best Challenge Score, accuracy and TPR and quite well TNR at the weight of 1.0. For the choice of the hidden feature size of the signals, we set the weight of the Discriminant Constraints to 1.0 and test the performance of 4 different hidden feature sizes of input signals, which are 16, 32, 64 and 128. From Table 2, we can see that using 64 as the hidden feature size of the signals inside our encoder, the model achieves best Challenge Score, accuracy and TPR.

The detailed 5-fold results on validation set. Table 3 reports the detailed performance on each fold. The performance variation in Challenge scores among the 5-fold is mainly caused by two reasons. First is the limited number of training records. Small number of training records results in the relatively large variance of each record, which may cause some difference in the distribution of each fold in the 5-fold training set. Second is the imbalance of true/false alarms and the imbalance of 5 different alarm types. There are 294 records with a true alarm and 456 records with a false alarm in the training set. What's more, considering the 5 different alarm types, the problem of imbalance is even more severe. For example, there are 131 ETC records with a true alarm and only 9 ETC records with a false alarm in the training set. The imbalance of classes in the dataset is always a challenging problem for deep learning models.

Some statistics on the dataset. Table 4 shows the number of records that each physiological signal occupies in the open access dataset. I, II, III, V, aVL, aVR, aVF and MCL are ECG signals. PPG, RESP and ABP are photoplethysmogram, respiration and arterial blood pressure respectively. Each record contains three or four signals, which are two leads of ECG and one or two supplementary signals as ABP, PPG or RESP.
