# Supplementary figures and images for: A contrastive learning approach for ICU false arrhythmia alarm reduction
Source: Sci Rep. 2022 Mar 18;12:4689. doi: 10.1038/s41598-022-07761-9 (PMC8933571; doi:10.1038/s41598-022-07761-9)

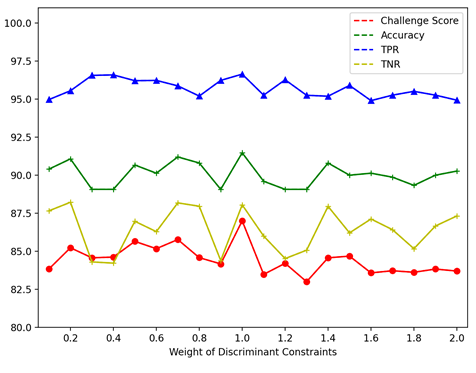

Supplement: Supplementary file 2 — Supplementary Figure 1. [file 41598_2022_7761_MOESM2_ESM.png]
